# High-Speed and Broadband InGaAs/InP Photodiode with InGaAsP Graded Bandgap Layers

**DOI:** 10.3390/s25092841

**Published:** 2025-04-30

**Authors:** Guohao Yang, Tianhong Liu, Jinping Li, Baile Chen, Cunzhu Tong

**Affiliations:** 1 Key Laboratory of Luminescence Science and Technology, Chinese Academy of Sciences, Changchun 130033, China; yangguohao17@mails.ucas.ac.cn (G.Y.); liutianhong22@mails.ucas.ac.cn (T.L.); tongcz@ciomp.ac.cn (C.T.); 2 State Key Laboratory of Luminescence and Applications, Changchun Institute of Optics, Fine Mechanics and Physics, Chinese Academy of Sciences, Changchun 130033, China; 3 University of Chinese Academy of Sciences, Beijing 100049, China; 4 School of Information Science and Technology, ShanghaiTech University, Shanghai 201210, China; chenbl@shanghaitech.edu.cn

**Keywords:** broadband, photodiode, graded bandgap layer, InGaAs/InP

## Abstract

This study presents the development of a high-speed, broadband InGaAs/InP photodiode suitable for advanced sensing and optical detection applications across the critical wavelength range of 850–1550 nm. By employing an InAlAs window layer to replace conventional InP, the device significantly improves sensitivity at 850 nm. Additionally, the substitution of traditional GaAs-based materials with InGaAs enhances responsivity and reduces carrier transit times, enabling precise, high-speed signal detection. The introduction of InGaAsP graded bandgap layers (GBLs) further improves device reliability and reduces absorption losses associated with defects, thus enhancing overall sensing performance. The fabricated photodiode, featuring an active area diameter of 35 µm, achieves high bandwidths of 20 GHz, 15 GHz, and 15.5 GHz at 850 nm, 1310 nm, and 1550 nm, respectively, along with responsivities of 0.5 A/W, 0.72 A/W, and 0.64 A/W. These characteristics make the device well suited for integration into multi-wavelength optical sensing systems, broadband photonic sensors, and high-speed optical communication platforms.

## 1. Introduction

The development of high-speed, broadband InGaAs/InP photodiodes (PDs) with a wide spectral response from 850 nm to 1550 nm is crucial for advanced optical sensing and data communication applications. This spectral range covers essential optical communication windows, including short-reach multimode fiber systems at 850 nm, intermediate-range networks at 1310 nm, and minimal-loss, long-haul fiber links at 1550 nm. A single photodiode capable of efficiently detecting multiple wavelengths simplifies optical sensor integration, supports flexible network configurations, and reduces overall system complexity and costs by eliminating multiple-wavelength-specific detectors. Furthermore, achieving high-speed detection with low junction capacitance and broad spectral responsivity directly addresses the growing demands for sensors and receivers used in next-generation optical networks, integrated photonic systems, and wavelength-division multiplexing sensing platforms [1,2].

Traditional GaAs-based photodiodes have been extensively used for detection near 850 nm. For instance, Wu et al. demonstrated an InGaP/GaAs photodiode achieving a maximum bandwidth of 9.7 GHz at 850 nm [3], while Bimberg et al. developed a GaAs/AlGaAs-based photodiode exhibiting approximately 13 GHz bandwidth and a responsivity of 0.5 A/W at 850 nm [4,5]. In addition to traditional PDs, Chen et al. achieved bandwidths of 28 GHz and 46 GHz for 34 μm and 20 μm diameter devices, respectively, using the inductive peaking method. In addition to traditional PDs, Long et al. achieved bandwidths of 28 GHz and 46 GHz for 34 μm and 20 μm diameter devices, respectively, using the inductive peaking method. Nevertheless, this resulted in a reduced responsivity of 0.44 A/W [6].

However, GaAs-based material exhibits limitations in terms of absorption efficiency and electron drift velocity, particularly at longer wavelengths like 1310 nm and 1550 nm. To overcome these challenges, the InGaAs/InP material system provides significant advantages, offering enhanced absorption properties and faster electron drift velocity over a broad wavelength range, making it ideal for broadband, high-speed photodiodes [7,8]. Such advantages enable enhanced sensitivity and rapid response times, crucial for high-speed optical sensing and data communication applications. Nevertheless, conventional InGaAs/InP photodiodes face challenges in optimizing high-speed performance at shorter wavelengths due to substantial absorption losses induced by interface defects at the InP window layer [9,10,11].

To address these challenges, Shi et al. utilized an InAlAs window layer coupled with an InAlGaAs graded bandgap layer (GBL), effectively reducing surface absorption losses, achieving a bandwidth of 14 GHz at 850 nm [12]. Furthermore, Wang et al. enhanced absorption using a distributed Bragg reflector (DBR) mirror, which shortened the absorption layer thickness, and achieved a bandwidth of 27.5 GHz with a responsivity of 0.5 A/W [13]. However, the aluminum content in InAlGaAs introduces lattice mismatches and associated defects, negatively impacting quantum efficiency and bandwidth performance. To further enhance photodiode performance, especially for broadband sensing applications, the InGaAsP material was introduced as the graded bandgap layer. InGaAsP significantly improves lattice compatibility with InP substrates, reducing defect-induced absorption losses and enhancing material quality. This structural refinement leads to optimized photodiodes, demonstrating significantly improved responsivity and bandwidth across the critical 850–1550 nm range. The PD achieves a bandwidth of 20 GHz with a responsivity of 0.5 A/W at 850 nm, directly meeting the performance requirements of advanced sensor systems.

## 2. Methods

Defects at the InP-air interface cause strong light absorption, reducing responsivity and bandwidth. A p-doped InAlAs layer is used to suppress this loss. However, material defects from the aluminum content in InAlGaAs GBL significantly lower responsivity and bandwidth. Replacing the GBL with InGaAsP, which offers better lattice matching to the InP-based material, effectively mitigates performance degradation in speed and quantum efficiency caused by material defects.

**Figure 1:**
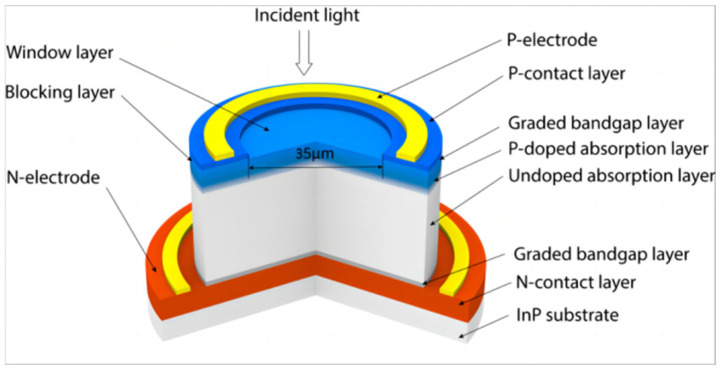
Schematic cross-sectional view of the InGaAs/InP photodiode with InGaAsP graded bandgap layers.

The structure of the InGaAs/InP photodiode is illustrated in Figure 1. Epitaxial layers were sequentially grown on an InP substrate, consisting of an InP n-contact layer, an InGaAsP GBL, an undoped InGaAs absorption layer, a p-doped InGaAs absorption layer, another InGaAsP GBL, an InP blocking layer, an InAlAs window layer, and an InGaAs p-contact layer. The device features a 300 nm p-doped graded absorption layer and a 4 μm undoped InGaAs absorption layer. The p-doped absorption layer absorbs incident light and creates a concentration gradient to accelerate electron diffusion, while the thick undoped layer leverages InGaAs’s superior electron transport to ease the trade-off between resistance-capacitance (RC) delay and carrier transit time.

**Figure 2:**
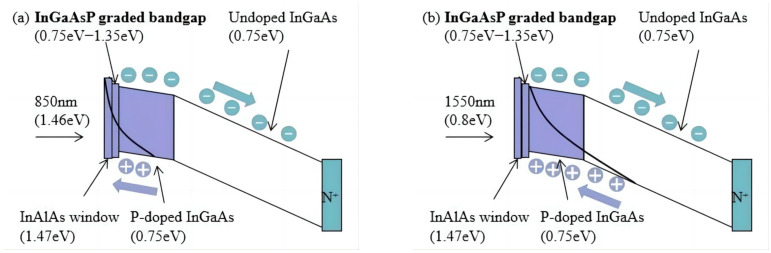
Schematic of the photodiode band structure under (**a**) short wavelength (850 nm) and (**b**) long wavelength (1550 nm) excitations.

At 850 nm, InGaAs features a high absorption coefficient, enabling the p-doped absorption layer to capture over 99% of incident light. As depicted in Figure 2a, electrons rapidly traverse the undoped absorption region at high velocity toward the n-electrode, resulting in a uni-traveling-carrier (UTC) photodiode configuration [14]. Under these conditions, nearly all absorption occurs within the p-doped layer, effectively eliminating the slower hole drift current in the undoped absorption layer, thereby enhancing device response speed. Conversely, at wavelengths of 1310 nm and 1550 nm, partial absorption occurs within the undoped absorption layer, as illustrated in Figure 2b. This process generates drift currents involving both electrons and holes, with the overall transit time dominated by the slower hole drift [15]. Consequently, the bandwidth at 1310 nm and 1550 nm is theoretically lower compared to that at 850 nm.

## 3. Device Fabrication

Based on the device structure detailed in Table 1, an epitaxial wafer was grown on a semi-insulating InP substrate via metal–organic chemical vapor deposition.

Initially, the epitaxial wafer was sequentially cleaned using acetone, isopropyl alcohol (IPA), and deionized water for 5 min each. A negative photoresist (Futurrex NR9-3000) was spin-coated onto the wafer surface at 3000 rpm, resulting in a thickness of approximately 2 µm. Photolithography was then performed to define electrode patterns. Subsequently, electron-beam evaporation (EBE, Winter WINVAC 400 L) was employed to deposit ring-shaped p-metal electrodes composed of Ti/Pt/Au (40 nm/20 nm/300 nm), leaving a gap on the ring to facilitate subsequent lift-off. The lift-off process involved immersion in acetone heated at 50 °C for 20 min to remove the photoresist. Following this step, alloying was conducted under a nitrogen atmosphere at 380 °C.

Mesa structures were etched using inductively coupled plasma (ICP, Oxford Plasmalab 100-ICP 180) with a gas mixture of Cl_2_, CH_4_, H_2_ and Ar. The InGaAs contact layer was selectively removed via ICP etching, exposing the underlying InAlAs window layer. Subsequently, a 300 nm Si_3_N_4_ passivation layer was deposited using plasma-enhanced chemical vapor deposition (PECVD, Oxford Plasmalab 100), and electrode windows were opened by ICP etching. The n-metal electrodes, consisting of Ni/AuGe/Ni/Au (20 nm/30 nm/20 nm/300 nm), were deposited on the exposed n-contact layer using EBE.

Benzocyclobutene (BCB, Cyclotene 4026) was spin-coated as an insulating layer to planarize mesa structures, electrically isolate the device surface, and effectively minimize crosstalk between metal electrodes. The BCB was cured under a nitrogen atmosphere at 250 °C for one hour, forming a stable three-dimensional structure with excellent electrical, mechanical, and thermal properties, ensuring device reliability and compatibility with subsequent processes. Windows for electrode exposure were then etched using reactive ion etching (RIE, Trion Phantom 3) with O_2_ and CF_4_ gases. In this process, O_2_ served as the primary etchant, while CF_4_ formed a fluorocarbon polymer layer on the photoresist surface, enhancing etch selectivity by protecting the resist.

Finally, coplanar electrodes connecting the p- and n-metal electrodes and pad electrodes for probing were deposited via EBE. The wafer was thinned using alumina (Al_2_O_3_) powder and water on a glass disk rotating at approximately 100 rpm until reaching a thickness between 150 nm and 200 nm. The thinned wafer was subsequently polished on a velvet polishing pad at approximately 150 rpm until a mirror finish was achieved, followed by a final rinse with deionized water for 10 min to remove residual contaminants.

**Figure 3:**
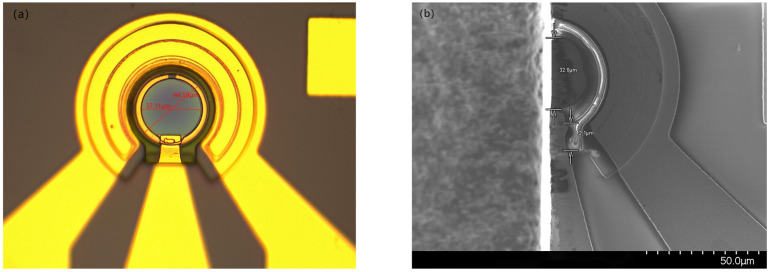
Images of the fabricated InGaAs/InP PD (**a**) under an optical microscope; (**b**) under a scanning electron microscope.

The fabricated devices were examined using optical microscopy and scanning electron microscopy (SEM), as illustrated in Figure 3. The inspection showed that the photosensitive area was smooth and uniform, with clear and intact electrode patterns, indicating a stable and well-established fabrication process without noticeable defects or contamination.

## 4. Results and Discussion

**Figure 4:**
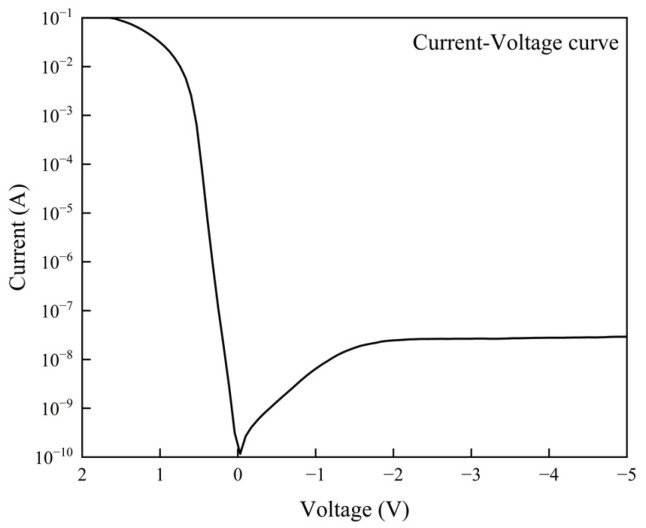
Current–voltage characteristics of the photodiode.

The current–voltage (I-V) characteristics, capacitance–voltage (C-V) characteristics and responsivity were measured using a semiconductor parameter analyzer (Keithley 4200A-SCS). The I-V characteristics are shown in Figure 4. The dark current of the PD stabilizes around 25 nA at approximately −2 V and reaches 27 nA at −3 V. The stable dark current indicates that the device exhibits minimal breakdown and leakage currents.

**Figure 5:**
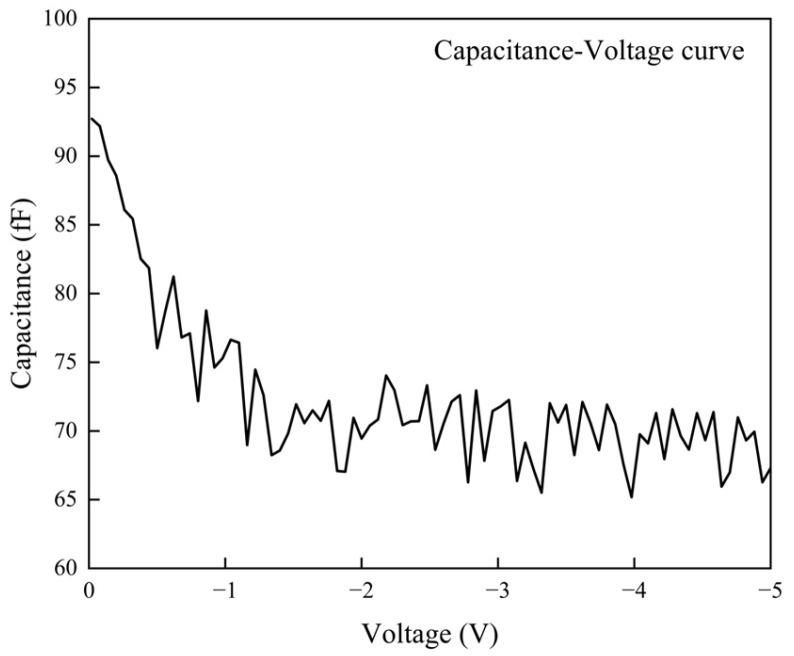
Capacitance–voltage characteristics of the photodiode.

The C-V characteristics are shown in Figure 5. After a bias of −1 V, the capacitance stabilizes, indicating that the depletion region is nearly fully depleted, and the device enters its operating state. At this point, the stable capacitance level of the PD is approximately 70 fF. The low dark current and capacitance confirm the success of the device fabrication process.

As shown in Table 2, the fabricated device achieved responsivities of 0.5, 0.72, and 0.64 A/W at 850, 1310, and 1550 nm, respectively, with corresponding quantum efficiencies of 73.0%, 68.1%, and 51.3%. This performance is comparable to that of state-of-the-art GaAs-based 850 nm photodiodes [4], which feature responsivities of 0.5–0.6 A/W. The high quantum efficiency demonstrates that the InAlAs window layer effectively prevents severe degradation caused by defects at the InP-air interface, ensuring most incident light is absorbed within the device. Additionally, in contrast to the substantial responsivity degradation observed in InAlGaAs GBLs (0.5 A/W vs. 0.25 A/W) due to material defects [12], InGaAsP GBLs offer superior lattice matching with the InP-based material, thereby improving overall device performance.

**Figure 6:**
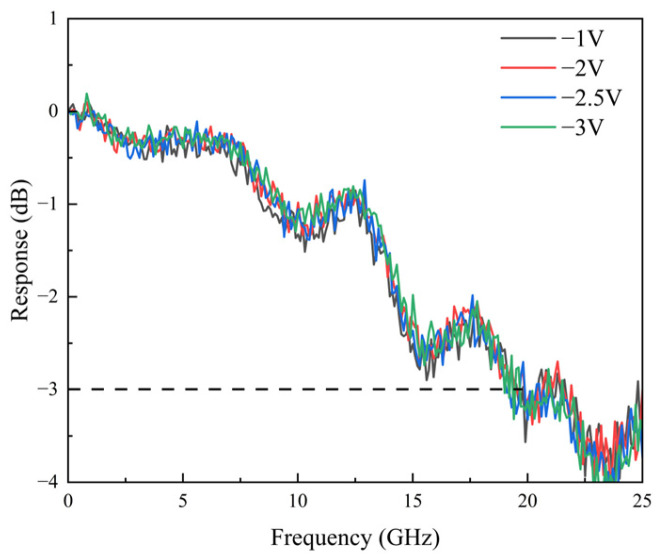
The measured OE response of the photodiode under 0.2 mA at 850 nm.

The frequency response was measured using a Lightwave component analyzer (LCA, KEYSIGHT N4373D) equipped with a performance network analyzer (PNA, KEYSIGHT N5227A). As illustrated in Figure 6, the device’s optical-electrical (OE) response at 850 nm was evaluated under varying bias voltages with an input optical power of 0.4 mW, and the maximum bandwidth reached 20 GHz within the optimal operating range (−1 to −3 V) [16]. This performance notably exceeds the 13 GHz bandwidth of GaAs-based photodiodes [4], demonstrating the promise of InP-based materials for high-speed photodetection at 850 nm. In the optimal operating range, the electron drift velocity in the undoped absorption layer remains nearly constant; thus, the device bandwidth shows minimal variation with reverse bias voltage. Compared with the 3 dB bandwidth reported for InAlGaAs GBL (14 GHz vs. 20 GHz) [12], the InGaAsP GBL minimizes bandwidth loss attributed to material defects [17].

**Figure 7:**
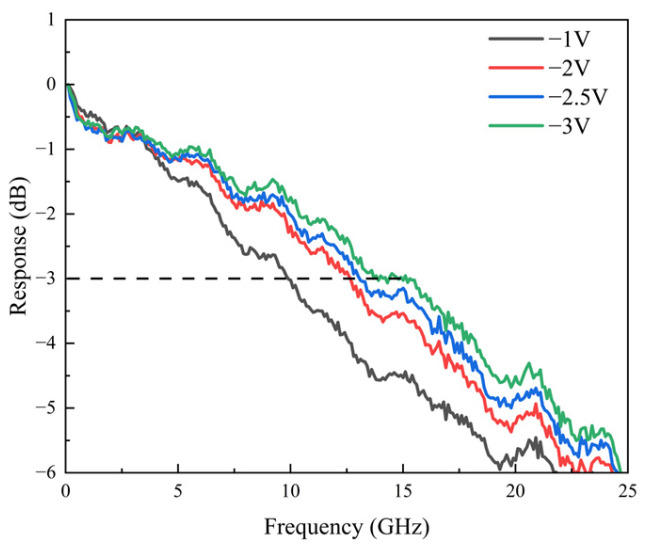
The measured OE response of the photodiode under 0.2 mA at 1310 nm.

As shown in Figure 7, the device’s OE response at 1310 nm with an input optical power of 0.3 mW attains a maximum bandwidth of 15 GHz at −3 V. Unlike at 850 nm, where the bandwidth depends solely on electron transport, part of the incident light at 1310 nm is absorbed in the undoped absorption layer, generating both electron and hole drift currents. The slower hole drift current constrains the device bandwidth. From the frequency response images, it is evident that as the reverse bias voltage increases, the hole drift velocity improves, resulting in higher bandwidth.

**Figure 8:**
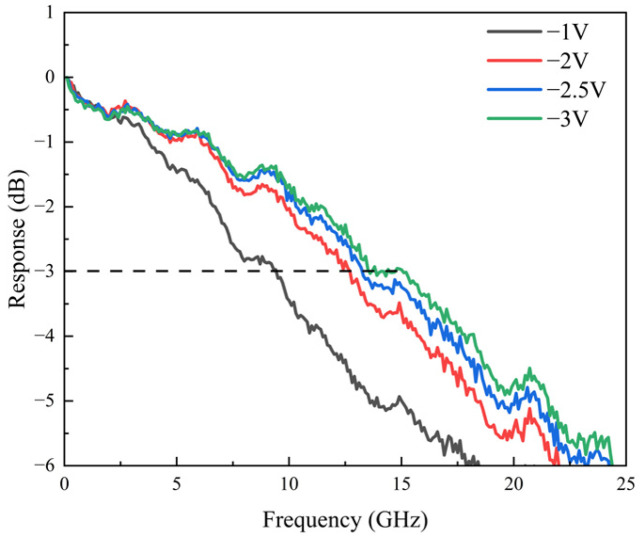
The measured OE response of the photodiode under 0.2 mA at 1550 nm.

Figure 8 illustrates the OE response of the device at 1550 nm with an optical power of 0.3 mW, where the maximum bandwidth reaches 15.5 GHz at −3 V. Although this is lower than the 25 GHz bandwidth reported for InP photodiodes, the present device supports a broader wavelength response and features a larger mesa diameter (35 µm vs. 21 µm), which enhances optical coupling and offers a wider dynamic range.

The bandwidth of a photodiode is primarily determined by the carrier transit time and the RC delay time. The total 3 dB bandwidth can be expressed as follows:

(1) $$\frac{1}{f_{3db}^{2}}=\frac{1}{f_{tr}^{2}}+\frac{1}{f_{RC}^{2}}=T_{tr}^{2}+T_{RC}^{2}.$$

The carrier transit time can be expressed as follows:

(2) $$T_{tr}=d_{diff}^{2}/D+d_{drift}/v_{drift}.$$

Here, *d_diff_* and *d_drift_* represent the thicknesses of the diffusion and drift layers, respectively; D represents the carrier diffusion coefficient; and *v_drift_* denotes the carrier drift velocity. In this structure, the drift length significantly exceeds the diffusion length; therefore, only the drift-induced delay is considered. According to relevant literature, at 850 nm, the electron velocity is taken as 1.5 × 10^7^ cm/s, while at 1310 nm and 1550 nm, the average drift velocity is approximately 5 × 10^6^ cm/s.

The RC delay time can be expressed as follows:

(3) $$T_{RC}=2\pi (R_{l}+R_{c})(C_{j}+C_{p}).$$

**Figure 9:**
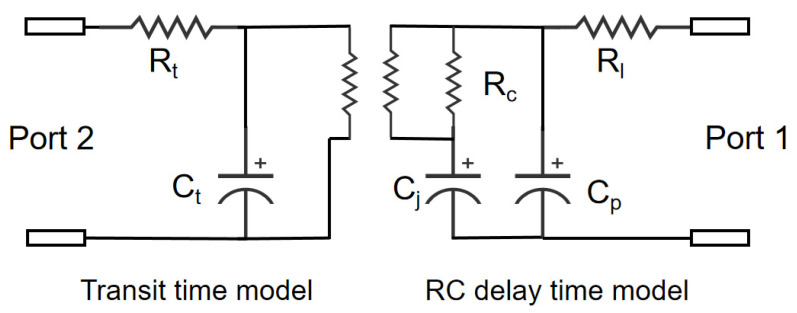
The equivalent circuit model of InGaAs/InP photodiode.

Here, *R_l_* represents the 50-ohm load resistance, *R_c_* is the contact resistance, *C_j_* denotes the junction capacitance, and *C_p_* corresponds to the parasitic capacitance. The equivalent circuit model is shown in Figure 9, and the simulation data are provided in Table 3.

**Figure 10:**
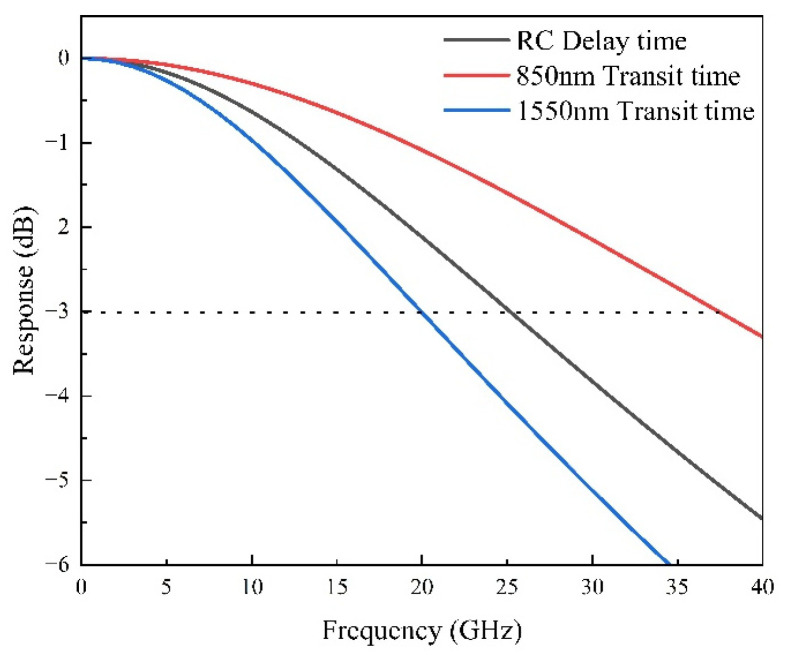
Simulated frequency response of RC delay time and transit time under short and long wavelength excitations.

As illustrated in Figure 10, the RC bandwidth is approximately 25 GHz and serves as the primary limiting factor for the bandwidth at the short wavelength of 850 nm. At 850 nm, the transit bandwidth is approximately 37.5 GHz, primarily due to the high absorption coefficient of InGaAs at this wavelength. The strong absorption leads to nearly complete absorption within the p-doped absorption layer. Consequently, only electrons are present as carriers in the depleted undoped absorption layer, and their drift velocity significantly exceeds that of holes, reducing the overall transit time. Incorporating the RC and transit bandwidths into Equation (1) yields a total calculated bandwidth of 20.8 GHz at 850 nm, closely aligning with the experimentally measured 20 GHz.

At 1550 nm, due to a lower absorption coefficient, the photodiode absorbs approximately 70% of the incident light within a 1.5 µm absorption layer (consisting of a 0.3 µm p-doped layer and a 1.2 µm undoped layer). Both electrons and holes are present within the depletion region, with the slower hole drift significantly limiting the transit bandwidth to around 20 GHz. This is notably lower compared to the bandwidth at shorter wavelengths, thus becoming the predominant limitation for the photodiode’s bandwidth at longer wavelengths. Substituting these conditions into Equation (1) provides an estimated total bandwidth of roughly 15.5 GHz at 1550 nm. The simulation results closely match experimental data, confirming the effectiveness and reliability of the current design approach.

Compared to devices utilizing InAlGaAs GBLs, our device exhibited reduced responsivity (0.64 A/W vs. 0.9 A/W) and lower bandwidth (15.5 GHz vs. 22 GHz) at 1550 nm [12]. This decrease in responsivity could be due to the P-doped absorption layer being too thin, leading to more holes entering the depletion region, causing severe space charge effects that hinder further current increase. Therefore, the thickness ratio between the p-doped and undoped absorption layers should be further optimized to improve the photodiode’s response speed at 1550 nm.

## 5. Conclusions

In conclusion, a high-speed, broadband photodiode based on the InGaAs/InP material system has been successfully demonstrated. By employing InAlAs as the window layer instead of InP and adopting InGaAs/InP in place of conventional GaAs-based structures, significant improvements in bandwidth and responsivity at 850 nm were achieved. Furthermore, the introduction of InGaAsP graded bandgap layers enhanced epitaxial quality and lattice compatibility, effectively reducing absorption losses and improving device performance across a broad spectral range. The fabricated photodiode, with an active area of 35 µm, exhibited measured bandwidths of 20 GHz, 15 GHz, and 15.5 GHz at 850 nm, 1310 nm, and 1550 nm, respectively, along with responsivities of 0.5 A/W, 0.72 A/W, and 0.64 A/W.

The wavelengths of 850 nm, 1310 nm, and 1550 nm are recognized as standard optical communication wavelengths, covering short-, medium-, and long-distance communication. By utilizing a single photodiode that can operate across these wavelength ranges, the need for different photodiodes is eliminated, reducing system complexity and cost. Additionally, these results demonstrate the strong potential of the photodiode that operates at 850 nm–1550 nm for both high-speed optical communication and broadband optical sensing applications, such as LiDAR, optical coherence tomography (OCT), and integrated photonic sensor networks, while providing flexibility and adaptability for use in optical fiber sensors, spectral analysis, and environmental monitoring.

## Figures and Tables

**Table 1:** Structure of the InGaAs/InP photodiode with InGaAsP graded bandgap layers.

Layer	Material	Thickness (nm)	Doping (cm^−3^)
P-contact layer	In_0.53_Ga_0.47_As	50	2 × 10^19^
Window layer	In_0.52_Al_0.48_As	50	1 × 10^19^
Blocking layer	InP	50	2 × 10^18^
Graded bandgap layer	In_0.65_Ga_0.35_As_0.3_P_0.7_	20	2 × 10^18^
	In_0.53_Ga_0.47_As_0.5_P_0.5_	20	2 × 10^18^
P-doped absorption layer	In_0.53_Ga_0.47_As	300	2 × 10^18^–1 × 10^17^
Undoped absorption layer	In_0.53_Ga_0.47_As	4000	1 × 10^15^
Graded bandgap layer	In_0.53_Ga_0.47_As_0.5_P_0.5_	20	1 × 10^15^
	In_0.65_Ga_0.35_As_0.3_P_0.7_	20	1 × 10^15^
N-contact layer	InP	500	1 × 10^19^

**Table 2:** Photocurrent responses at various wavelengths under a 2.5 V bias and at 0.5 mW.

Wavelength (nm)	Test	Photocurrent (mA)	Responsivity (A/W)
850	1	0.249	0.498
850	2	0.241	0.482
850	3	0.241	0.482
1310	1	0.362	0.724
1310	2	0.341	0.682
1310	3	0.345	0.690
1550	1	0.321	0.642
1550	2	0.311	0.622
1550	3	0.307	0.614

**Table 3:** Simulation parameters of the photodiode [8,18].

Parameters	850 nm	1550 nm
Absorption coefficient	3.5 μm^−1^	0.8 μm^−1^
Average electron drift velocity	1.5 × 10^7^ cm/s	1.9 × 10^7^ cm/s
Average hole drift velocity		6 × 10^6^ cm/s
Active diameter	35 μm	35 μm
Load resistance	50 Ω	50 Ω
Contact resistance	40 Ω	40 Ω
Junction capacitance and parasitic capacitance	70 fF	70 fF

## Data Availability

Data are contained within the article.
